# Karst tiankengs as refugia for indigenous tree flora amidst a degraded landscape in southwestern China

**DOI:** 10.1038/s41598-017-04592-x

**Published:** 2017-06-26

**Authors:** Yuqiao Su, Qiming Tang, Fuyan Mo, Yuegui Xue

**Affiliations:** College of Life Sciences, Guangxi Normal University, Guilin, 541006 Guangxi China

## Abstract

We conducted floristic and community analyses to compare the floristic composition, forest structure, taxonomic richness, and species diversity between two tiankeng (large doline, or sinkhole) habitats and two outside-tiankeng habitats of forest fragments in a degraded karst area in southwestern China. We found remarkably higher taxonomic richness in the tiankeng habitats than in the outside-tiankeng habitats at the species, generic, and familial levels. The inside-tiankeng habitats had higher floristic diversity but lower dominance. The remarkably higher uniqueness at all taxonomic levels and the much larger tree size in the two tiankeng habitats than in the outside-tiankeng habitats demonstrated the old-growth and isolated nature of the tiankeng flora. Plot-scale species richness, Shannon-Wiener index, Pielou’s evenness, and Berger-Parker dominance significantly differed across habitats. Heterogeneity in floristic composition at the species, generic, and familial levels was extremely significant across habitats. In pairwise comparisons, except for the Chuandong Tiankeng-Shenmu Tiankeng pair, all the pairs showed significant between-habitat heterogeneity in floristic composition. Our results suggest that as oases amidst the degraded karst landscape, tiankengs serve as modern refugia that preserve old-growth forest communities with their rich floristic diversity, and can provide a model for habitat conservation and forest restoration in that area.

## Introduction

Biodiversity loss, habitat fragmentation, and land degradation characterised by soil erosion, lower fertility, and rocky desertification are the typical features of mountainous karst landscapes^1–3^. The karst landforms are widespread but with restricted distributions in the world^4, 5^. While the karst landforms are associated with splendid landscapes and picturesque views, they are commonly regarded as an ecologically vulnerable system^6, 7^. In China, karst landforms occur mainly in southwestern China’s Guangxi, Guizhou, and Yunnan provinces. During the past twenty years, a geographic wonder called tiankeng has been discovered in the karst areas of southwestern China, typically in Leye county of Guangxi, and revealed to the outside world. A tiankeng is a unique type of large doline, or sinkhole, as defined in Chinese and English literature^8–12^, and it can be translated literally from the Chinese as *heavenly pit*. The rapidly developing tourism industry and worldwide cave exploration efforts have driven the discovery of karst tiankengs. Current, although limited, investigations from excursions to this area have revealed that the tiankengs contain pristine old-growth forest^8, 13^, thus making them oases amidst the degraded karst landscape. We hypothesised that these tiankengs might serve as refugia for the once rich biotic diversity in this area.

Investigating the floristic composition and structure of forest communities that occur in special landforms and terrains, such as tiankengs, or dolines, will help elucidate the historical distribution of plant communities and their succession, shift with environmental change, and plant phylogeography. Dolines in different parts of the world are morphologically different and may have different names^10, 12^. While the large collapsed doline in southwestern China has been given a term called tiankeng, a type of collapsed doline in Mexico is called Rejolladas^14^. Similarly, dolines are called Japage in Croatia^15^ and Minyé in Papua New Guinea^11^. Recent studies indicated that dolines in Hungary play a key role in preserving different groups of species, and many endangered vascular plant species occur there^16–19^. Several other studies on the vegetation and environment relations also suggested that dolines are rich in plant and animal lives^20–22^ and some reported new taxa found in dolines^23, 24^.

As a special type of negative terrain, a tiankeng is a large collapsed doline at least 100 m deep and wide, commonly surrounded by vertical cliffs and inaccessible to humans^12^. The pristine forest inside a tiankeng mirrors the natural geological and ecological processes in the region. Due to tiankengs’ inaccessibility, the studies that have been conducted are limited to general scientific surveys, geological investigations, and touristic explorations^12, 25^. A few studies on plant resources, Chinese traditional herbs^13, 26^, and soil pollution monitoring^27–29^ have recently been reported, but we know little of the composition, diversity, and structure of the “underground” vegetation inside the tiankengs. Our preliminary report on the forest community structure and diversity in Liuxing Tiankeng from this area^8^ may be the first published report on the ecological analysis of the forest community inside a tiankeng.

A good understanding of the forest communities that have been preserved in the tiankeng habitats will also help in the design of conservation strategies. The flora and forest community of tiankengs provide a model of a species pool for protecting the extant fragmented vegetation and restoring a species-rich forest ecosystem in the outside-tiankeng degraded landscape. In this study, we aimed to reveal the floristic composition and community structure of the tiankeng pristine forest compared to those of the outside-tiankeng forest fragments. To achieve this goal, we compared the floristic composition, taxonomic richness, tree size, and species diversity between two tiankeng habitats and two outside-tiankeng habitats in forest fragments of a degraded karst area in southwestern China.

## Results

### Floristic richness and abundance

**Figure 1:**
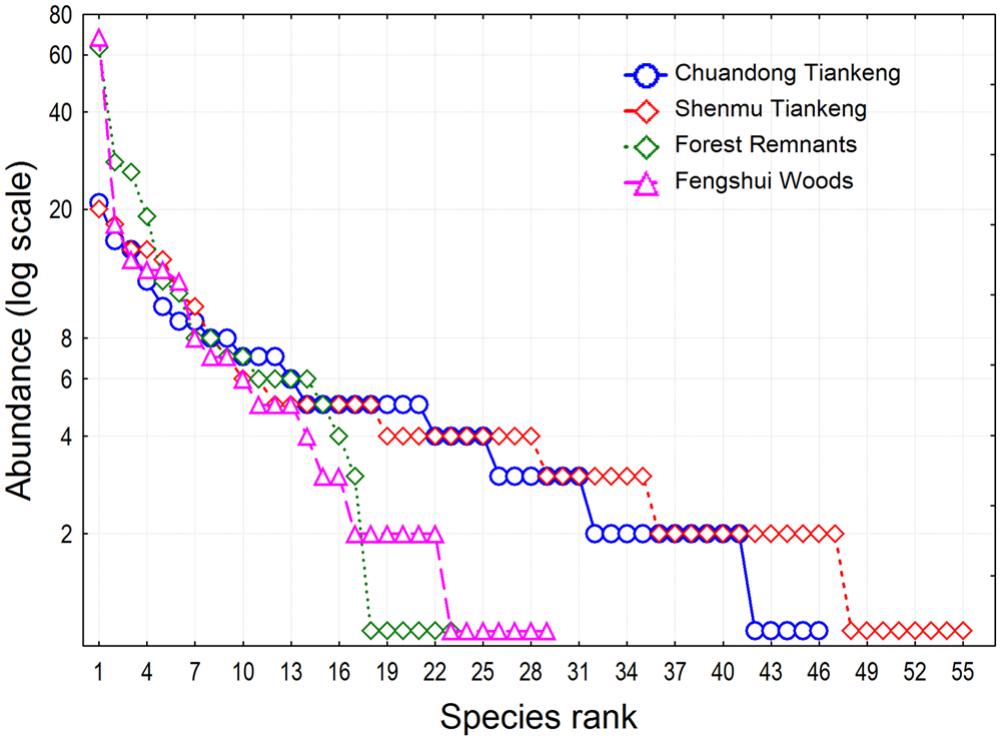
Rank abundance curves by species, showing number of species across habitats and the abundance and evenness patterns.

**Figure 2:**
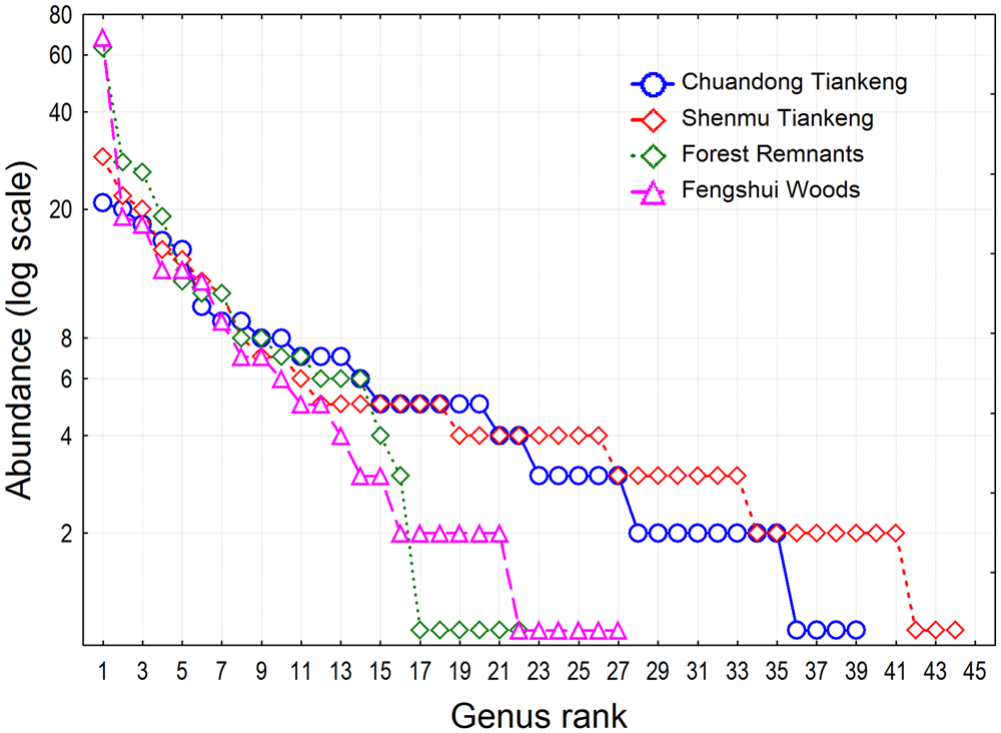
Rank abundance curves by genus, showing number of genera across habitats and the abundance and evenness patterns.

**Figure 3:**
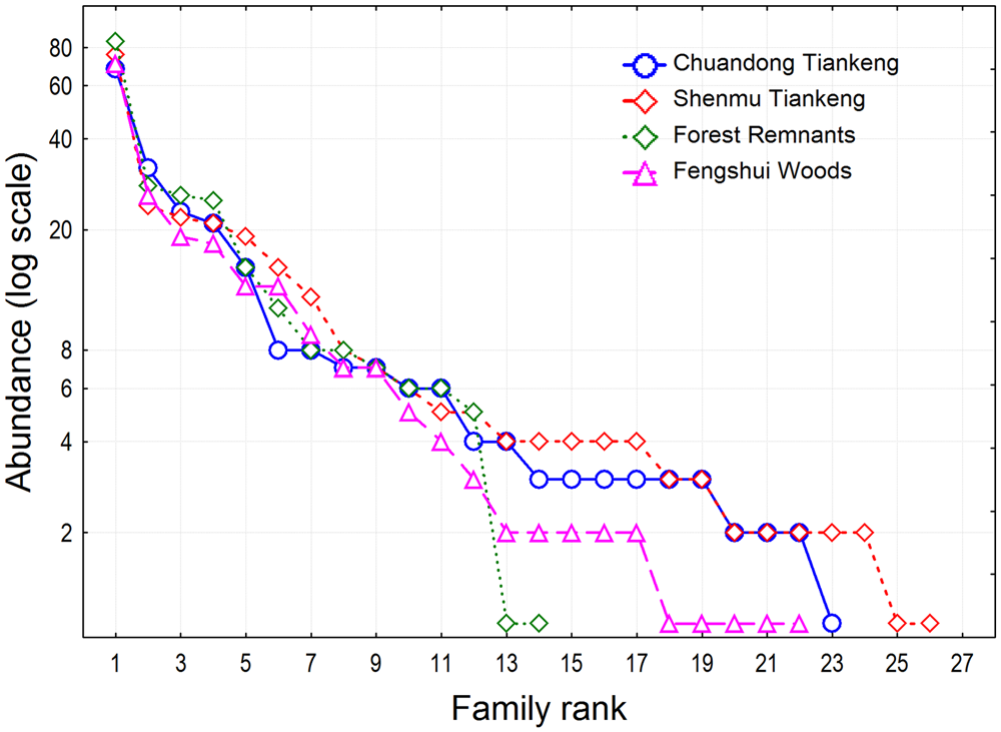
Rank abundance curves by family, showing number of families across habitats and the abundance and evenness patterns.

For tree stems ≥ 10 cm diameter at breast height (DBH), we recorded a total of 933 tree individuals of 96 species from 66 genera and 38 families in all four habitats (Supplementary Table 1; see the Methods section for the definition of the four habitats.). We found contrasting patterns in species composition and higher-taxa composition. The two tiankeng habitats had remarkably higher taxonomic richness at the species, generic, and familial levels than the two outside-tiankeng habitats. The species richness in the four habitats, Chuandong Tiankeng, Shenmu Tiankeng, Forest Remnants, and Fengshui Woods, was 46, 55, 23, and 29, respectively; the generic richness was 39, 44, 22, and 27, respectively; and the familial richness was 23, 26, 14, 22, respectively (Figs 1–3). The most dominant species in the four habitats included 21, 20, 63, and 68 tree individuals, respectively; the most dominant genus included 21, 29, 63, and 68 tree individuals, respectively; and the most dominant family had 68, 76, 84, and 71 individuals, respectively (Figs 1–3). The rank-abundance curves (diversity-dominance curves) for the two outside-tiankeng habitats were much steeper than those for the inside-tiankeng habitats, indicating that the outside-tiankeng habitats had higher floristic dominance but lower floristic diversity and evenness.

### Shared and unique taxa

**Figure 4:**
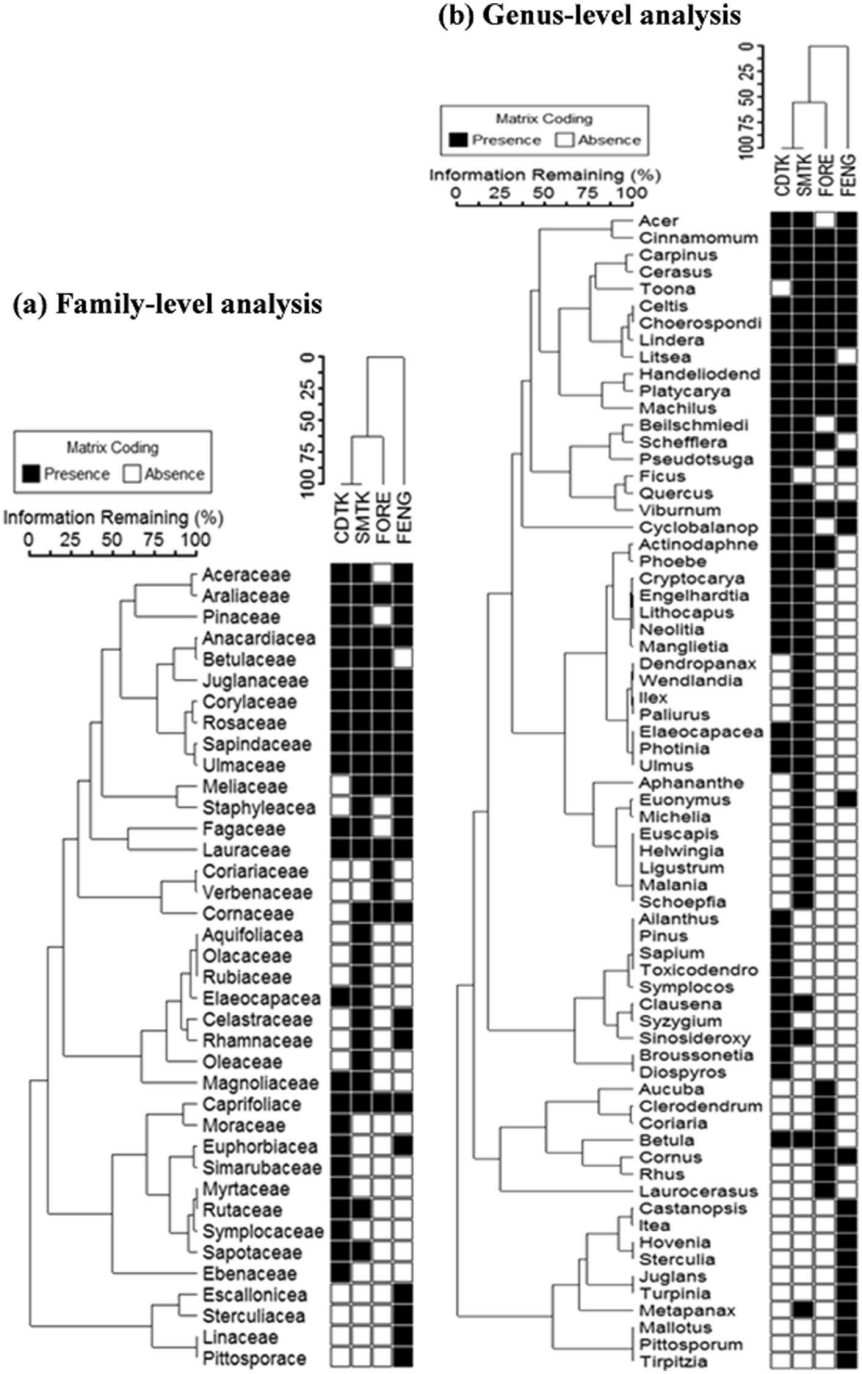
Two-way cluster dendrograms showing habitat associations and the groupings of similar floristic distribution at the levels of family (**a**) and Genus (**b**). Habitat code: CDTK = Chuandong Tiankeng; SMTK = Shenmu Tiankeng; FORE = Forest Remnants; FENG = Fengshui Woods.

A comparison of the shared and unique taxa between the tiankeng habitat type and the outside-tiankeng habitat type demonstrated the unique and ancient nature of the tiankeng flora. The inside-tiankeng habitats shared 19 families, 22 genera, and 13 species with the outside-tiankeng habitats. While 13 families, 31 genera, and 56 species, representing 41%, 58%, and 81%, respectively, of the total number of taxa occurring in the tiankeng habitats, were unique, only 4 families, 15 genera, and 27 species, accounting for 16%, 40%, and 68%, respectively, of the total number of taxa occurring in the outside-tiankeng habitats, were unique to the outside-tiankeng habitats (Table 1; see also Fig. 4 and Supplementary Figure S1). The remarkably higher uniqueness of the inside-tiankeng flora at all the taxonomic levels further demonstrated the primitive and isolated nature of the tiankeng flora and the degradation of the outside-tiankeng habitats.

**Table 1:** Floristic composition and the shared and unique taxa between inside-tiankeng and outside-tiankeng habitats.

Habitat	Family	Genus	Species
Inside Tiankeng			
Chuandong Tiankeng	23	39	46
Shenmu Tiankeng	26	44	55
Overall	32	53	69
Outside Tiankeng			
Forest Remnants	14	22	23
Fengshui Woods	22	27	29
Overall	25	37	40
Gamma diversity of the four habitats	38	68	96
Shared taxa between inside-Tiankeng and outside-Tiangkeng habitat types	19	22	13
Unique taxa to inside-Tiankeng type	13	31	56
Unique taxa to outside-Tiankeng type	4	15	27

### Tree size structure

**Figure 5:**
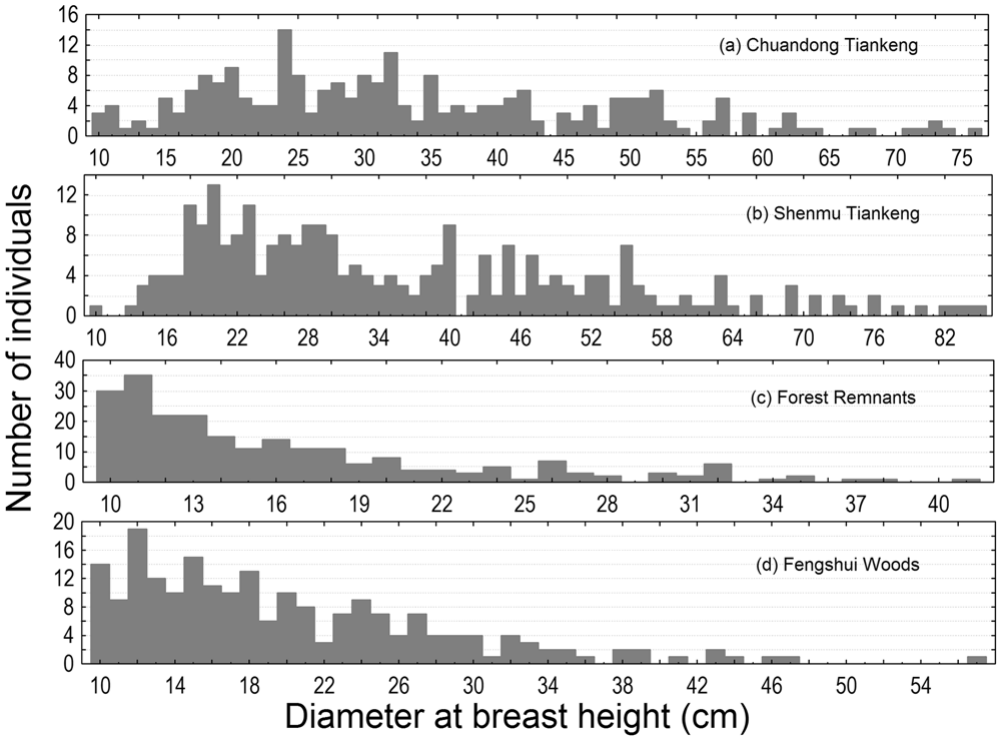
Histograms showing tree size distribution across habitats in the karst tiankeng landscape.

The inside-tiankeng habitats had many more large trees than the outside-tiankeng habitats. The largest tree occurring in each of the four habitats, Chuandong Tiankeng, Shenmu Tiankeng, Forest Remnants, and Fengshui Woods, had a maximum DBH of 76.0 cm, 85.0 cm, 41.2 cm, and 57.0 cm, respectively (Fig. 5). The number of large trees found in the tiankeng habitats was much greater than the number found in the outside-tiankeng habitats. The inside-tiankeng habitats had 267 large adult trees (DBH ≥ 30.0 cm), accounting for 54% of the total number of tree individuals occurring in the sampling plots there, whereas only 45 trees of such size were found in the sampling plots of the outside-tiankeng habitats, accounting for 10% of the total number of tree individuals occurring there (Fig. 5). The contrast in tree size structure between the inside-tiankeng and outside-tiankeng forest communities again showed the old-growth and isolated nature of the inside-tiankeng habitats.

### Species diversity

**Figure 6:**
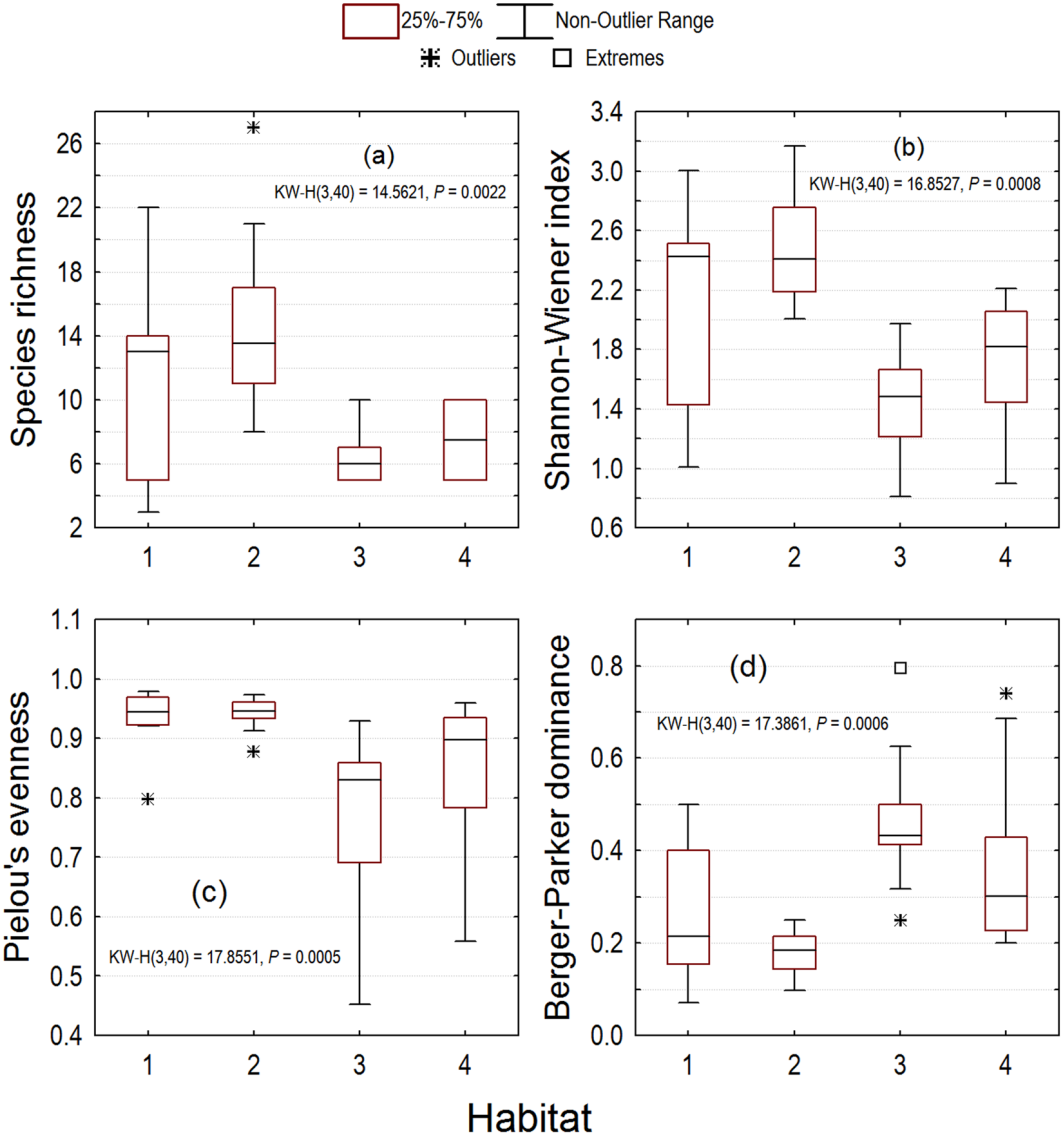
Plot-scale species richness and diversity across habitats in a karst tiankeng landscape. Habitat code: 1** = **Chuandong Tiankeng; 2 = Shenmu Tiankeng; 3 = Forest Remnants; 4 = Fengshui Woods.

We found significant differences in plot-scale species richness (Kruskal-Wallis test, *P* = 0.0022), Shannon-Wiener index (Kruskal-Wallis test, *P* = 0.0008), Pielou’s evenness (Kruskal-Wallis test, *P* = 0.0005), and Berger-Parker dominance (Kruskal-Wallis test, *P* = 0.0006) across habitats (Fig. 6). Species richness and the Shannon-Wiener index showed similar patterns, and the inside-tiankeng habitats had significantly higher overall species richness and diversity than the outside-tiankeng habitats. Pielou’s evenness showed different patterns from Berger-Parker dominance. Trees had a more even distribution with much smaller ranges in the forest communities of Chuandong Tiankeng and Shenmu Tiankeng than in the Forest Remnants and Fengshui Woods communities (Fig. 6c).

### Habitat heterogeneity

Multi-response permutation procedures (MRPP) presented overall and pairwise tests for significance of variation in taxonomic composition across habitats. By overall comparison, we found extremely significant differences in floristic composition across habitats at the familial, generic, and species levels (MRPP, *P* < 10^−5^). The highest within-habitat homogeneity (MRPP, *A = *0.102) and between-habitat heterogeneity (MRPP, *T* = −11.260) across habitats existed at the species level (Table 2). By pairwise comparison, all the pairs except the pair of Chuandong Tiankeng and Shenmu Tiankeng showed significant differences. Floristic composition did not differ between the Chuandong Tiankeng and Shenmu Tiankeng habitats at the familial (MRPP, *P* = 0.082), generic (MRPP, *P* = 0.094), and species (MRPP, *P* = 0.070) levels (Table 2).

**Table 2:** Multi-response permutation procedures (MRPP) for the taxonomic composition across habitats.

Habitats compared	Variance	Skewness	*T*	*A*	*P*
**Familial level:**					
Overall comparison	0.706	−0.593	−6.664	0.077	<10^−5^
Pairwise comparison					
CDTK vs. SMTK			−1.515	0.027	0.082
CDTK vs. FORE			−3.663	0.063	0.005
CDTK vs. FENG			−3.356	0.050	0.007
SMTK vs. FORE			−3.526	0.052	0.003
SMTK vs. FENG			−4.977	0.065	<0.001
FORE vs. FENG			−4.402	0.063	<0.001
**Generic level:**					
Overall comparison	0.581	−0.516	−7.822	0.073	<10^−7^
Pairwise comparison					
CDTK vs. SMTK			−1.390	0.021	0.094
CDTK vs. FORE			−3.848	0.055	0.002
CDTK vs. FENG			−4.257	0.057	0.002
SMTK vs. FORE			−4.296	0.054	<0.001
SMTK vs. FENG			−5.940	0.065	<10^−4^
FORE vs. FENG			−4.008	0.054	<0.001
**Species level:**					
Overall comparison	0.637	−0.585	−11.260	0.102	<10^−8^
Pairwise comparison					
CDTK vs. SMTK			−1.651	0.022	0.070
CDTK vs. FORE			−6.200	0.086	<10^−4^
CDTK vs. FENG			−6.805	0.091	<10^−4^
SMTK vs. FORE			−7.573	0.093	<10^−5^
SMTK vs. FENG			−7.965	0.087	<10^−5^
FORE vs. FENG			−4.012	0.051	<0.001

Definitions of the MRPP statistics: *A* is a measure of effect size and represents the within-group homogeneity; *T* is a statistic describing the separation between the groups; and *P* is the *P*-value for significance test of homogeneity using permutation. Habitat code: CDTK **=** Chuandong Tiankeng; SMTK = Shenmu Tiankeng; FORE = Forest Remnants; FENG = Fengshui Woods.

The within-habitat homogeneity and between-habitat heterogeneity across habitats were confirmed by the results from two-way cluster analysis at the familial, generic, and species levels. The two-way cluster dendrograms (Fig. 4 and Supplementary Figure S1) showed habitat associations with the taxonomic entities. The taxonomic entities with similar habitat specificity were clustered in a group with close membership, while the habitats associated with similar floristic distributions were grouped together. At all the taxonomic levels, Chuandong Tiankeng and Shenmu Tiankeng were grouped together with 100% information remaining. The Forest Remnants habitat as a close branch was fused into the Chuandong Tiankeng and Shenmu Tiankeng cluster at the familial and generic levels (Fig. 4), while at the species level, the Forest Remnant and Fengshui Woods were first grouped together into a cluster with 65% information remaining; then, this cluster was fused into a single group with the Chuandong Tiankeng and Shenmu Tiankeng cluster (Supplementary Figure S1).

## Discussion

Restoring the vegetation in the degraded karst areas is a major challenge for biodiversity conservation and regional development^30, 31^, since these areas have undergone longtime environmental degradation and biodiversity loss due to geological and climatic processes, as well as intense human activities^32, 33^. However, the recent discoveries of tiankengs (large dolines) in the karst area of southwestern China have shed light on this goal, because tiankeng habitats may have preserved the intact vegetation from a long–ago period. In this study, we conducted floristic and community analyses to compare the floristic richness, species diversity and forest structure between the subterranean tiankeng habitats and the outside-tiankeng Forest Remnants and Fengshui Woods using a plot sampling method. We found remarkably higher taxonomic richness in the tiankeng habitats than in the two outside-tiankeng habitats at the species, generic, and familial levels. The rank-abundance curves for the two outside-tiankeng habitats were much steeper than those for the inside-tiankeng habitats, indicating that the inside-tiankeng habitats had higher floristic diversity and evenness but lower dominance. The remarkably higher floristic uniqueness at all the taxonomic levels inside the tiankengs, compared to the outside-tiankeng habitats, demonstrated the primitive and isolated nature of the tiankeng flora and the degradation of the outside-tiankeng habitats.

Tree size distribution is the fundamental forest stand structure from which we can judge or model forest age and growth status^34–36^. The contrasting tree size structure between the inside-tiankeng and the outside-tiankeng forest communities again showed the old-growth and isolated nature of the inside-tiankeng habitats. Community analyses showed changes in species diversity attributes at the plot scale across habitats. We found significant differences in the plot-scale species richness, Shannon-Wiener index, Pielou’s evenness, and Berger-Parker dominance across habitats. MRPP indicated that heterogeneity in floristic composition at the species, generic, and familial levels was extremely significant for overall comparison across habitats. For pairwise comparison, except for the Chuandong Tiankeng-Shenmu Tiankeng pair, all the pairs showed significant between-habitat heterogeneity in floristic composition. Our results suggest that the tiankeng habitats serve as modern refugia that preserve old-growth forest communities with higher floristic diversity and predominantly much older trees than the degraded outside-tiankeng landscapes.

A refugium refers to an area in which climate and vegetation have remained relatively unchanged while areas surrounding it have changed markedly^37^. In contrast to the arid, degraded landscape outside the tiankengs, the humid microclimate inside them sustained a rich flora because of the underground river system in the cone karst area^10, 27^ and the relatively isolated habitat deep inside the earth. Unlike the traditional refugium concept, which describes plant diversity as having survived from the widespread Pleistocene glaciers on the earth^37^, tiankengs are oases amidst the degraded karst landscape. They preserve the modern flora, with its rich diversity at a local or regional scale, and thus serve as modern refugia that protect the indigenous flora from the land degradation and biodiversity loss caused by karstification and human disturbance. Although the term tiankeng has entered the international karst lexicon, there is still controversy about the relationship between tiankeng and doline^38, 39^. The geographical differences in the relationships between the vegetation and the morphogenesis of tiankengs and dolines are still to be explored, in order to determine the mechanisms driving tiankengs’ functionality as the refugia for the local biota. Our study of the tiankeng flora and forest communities in the karst areas in southwestern China will provide an important case study for the international karst and speleology research, biodiversity conservation in karst areas, and related biological resource studies.

The indigenous tree flora and vegetation in the area have been ruined and fragmented by longtime natural processes such as geological and climatic events as well as intense recent human activities. However, down in the tiankengs, the pristine forest with its rich diversity and old-growth structure has been preserved, thus providing a genetic pool of the indigenous flora and possibilities for scientific research and revegetation in the degraded karst areas. The tiankengs not only are the refugia for the richly diverse indigenous biota, but also serve as tourist attractions for the outside world. Tiankengs in Leye, Guangxi Zhuang Autonomous Region, have been discovered and revealed to the outside world only in the past 20 years, but they have attracted attention worldwide. The rapidly developing tourism industry has taken advantage of this area for its tourist value and made it a well-known tourist destination. Tourist activities in this area have created new perils for the tiankeng ecosystem; therefore, conservation efforts in this area should prioritise tiankeng habitats and their rich biodiversity.

## Methods

### Study area

Our study area was located in Leye county of Guangxi Zhuang Autonomous Region, southwestern China (106°10′–106°51′E, 24°30′–25°03′N). This area is on the southeast part of the Yunnan-Guizhou Plateau, which has a land surface characterised by cone karst terrain^10^. The area has a mid-subtropical monsoon climate with contrasting wet and dry seasons over the course of the year, a mean annual temperature of 16.6 °C and a mean annual precipitation of 1,400 mm^8^.

### Sampling design and plant census

Two inside-tiankeng habitats and two outside-tiankeng habitats were selected for our study. Chuandong Tiankeng (coded as CDTK) has an average depth of 175 m and a maximum depth of 312 m, with a horizontal dimension of 270 m × 370 m at the mouth, which opens onto a mountaintop^11^. Shenmu Tiankeng (coded as SMTK) has an average depth of 186 m and a maximum depth of 234 m, with a horizontal dimension of 340 m × 370 m at the mouth, which opens into the valley of the cone karst landscape^11^. Outside the tiankengs on the nearby land surface, no continuous forest vegetation was found, only sporadic forest fragments. These forest fragments fall into two categories: one with frequent human interventions, such as harvesting fuelwoods, medicinal herbs, wild vegetables, and other forest by-products, as well as natural disturbances from rocky desertification, and the other, commonly referred to as Fengshui Woods, found near human settlements with intentional and rigorous protection for shading, windbreak, and/or sacred purposes^40, 41^. In our study, the first category of forest fragments was coded as FORE (Forest Remnants), and the other was coded as FENG (Fengshui Woods). Thus, two inside-tiankeng habitats and two outside-tiankeng habitats were defined for our study.

Before we established plots for plant census, we made a three-day preliminary excursion to Chuandong Tiankeng, Shenmu Tiankeng, and the nearby outside-tiankeng land surface locations to gain a general knowledge of the forest communities both inside and outside the tiankengs. The preliminary survey focused on the habitat conditions and forest community physiognomy and helped locate the forested sites in each habitat for plot sampling. In each of the four habitats, we established five sampling plots, each containing two contiguous 20 m × 20 m quadrats for the census of trees. Such a plot dimension for tree sampling is appropriate and can be used in all the four habitats to make field data comparable, because some of the outside-tiankeng forest fragments are too small to accommodate more than two contiguous 20 m × 20 m quadrats. All trees ≥ 10 cm diameter at breast height (DBH) were tallied; tagged; identified to species; and recorded by species name, DBH, and tree height. DBH was measured with a diameter tape to the nearest 0.1 cm. Tree height was measured to the nearest 0.1 m with a measuring rod for trees ≤ 6.5 m and a clinometer (Suunto PM-5/1520, Finland) for trees > 6.5 m^42, 43^. Tree height data were not used in this study. The plant taxonomy and systematics follow the *Checklist of Guangxi Plants* ^44^.

### Data analysis

Many ecological studies focus on analyses at the species level. Here, apart from species analysis, we extended the statistical applications to higher-taxa analyses to comprise the generic and familial levels, because we intended to extract floristic patterns by comparing the forest communities of the inside-tiankeng habitats and the outside-tiankeng habitats. We constructed rank-abundance curves to elucidate floristic diversity and dominance patterns across habitats at the species, generic, and familial levels. The rank-abundance curve, also called the Whittaker plot, is a common statistical technique to plot abundance (or relative abundance) against an ordered taxonomic entity, which is ranked from the highest to the lowest abundance^45, 46^.

To visualize habitat association with taxonomic entities at the familial, generic, and species levels, we performed two-way cluster analysis on the plot × family, plot × genus, and plot × species datasets of abundance, using group average method with Bray-Curtis distance^47, 48^, to show habitat relationships and groupings of taxonomic entities with similar distribution patterns. Two-way cluster analysis simultaneously classifies habitats and visually depicts the ecological similarities or differences in or between clusters of taxonomic entities^47, 48^. Interspecific association can also be observed from species presence/absence distribution in a particular habitat or habitat type in the dendrogram generated by two-way cluster analysis.

To contrast differences in tree size across habitats, we plotted histograms of tree DBH by habitat. The histograms show the full spectra of the tree DBH distribution and give an impressive representation of the approximate stand age of each habitat, since DBH at community level is a good proxy variable for stand age, given similar forest types (e.g., all are natural hardwood forest.)^34, 35, 49^.

To assess differences in community patterns across habitats, we performed the Kruskal-Wallis test, a nonparametric alternative to analysis of variance (ANOVA), to test for significance of differences in plot-scale species richness, Shannon-Wiener diversity index, Pielou’s evenness, and Berger-Parker dominance. While species richness was represented by the number of species in a quadrat, Shannon-Wiener index, Pielou’s evenness, and Berger-Parker dominance were calculated using the following equations^46^, respectively:

1 $$H^{\prime} =-\sum {P}_{i}\,\mathrm{ln}\,{P}_{i}$$

2 $$E=H^{\prime} /\mathrm{ln}\,S$$

3 $${D}_{B-P}={N}_{{\rm{\max }}}/N$$

where *H*′ is the Shannon-Wiener index; *E* is Pielou’s evenness; *D*_*B*−*P*_ is Berger-Parker dominance; *S* is the number of species; *N* is the total number of individuals; *N*_max_ is the number of individuals of the most abundant species; and *P*_*i*_ is the relative abundance of the *i*th species, calculated as *P*_*i*_ = *n*_*i*_ */N*.

To evaluate variations in floristic composition at the familial, generic, and species levels across habitats, we performed multi-response permutation procedures (MRPP) on the plot × family, plot × genus, and plot × species datasets of abundance across habitats and made pairwise comparisons between habitats. MRPP is a nonparametric procedure for testing the hypothesis of no difference between two or more *a priori* groups of entities^47, 48^. The output of MRPP includes three major statistics: *T*, *A*, and *P*-value. *T* is a test statistic that describes the separation between groups, while *A* is a descriptor of within-group homogeneity, known as the “effect size”, or chance-corrected within-group agreement^47, 48^.

The rank-abundance curve analysis and the Kruskal-Wallis test were carried out with STATISTICA data analysis software system, version 8.0 (Statsoft, Inc. Tulsa, OK, USA), while two-way cluster analysis and MRPP were performed with PC-ORD, multivariate analysis of ecological data, version 6.0 (MjM Software, Gleneden Beach, OR, USA).

### Data Availability

The datasets generated during the current study are available from the corresponding author on reasonable request.

## Electronic supplementary material

Supplementary Table S1 & Figure S1
