# Supplementary material for: Karst tiankengs as refugia for indigenous tree flora amidst a degraded landscape in southwestern China
Source: Sci Rep. 2017 Jun 26;7:4249. doi: 10.1038/s41598-017-04592-x (PMC5484678; doi:10.1038/s41598-017-04592-x)
Supplement: Supplementary file 1 — Supplementary Table S1 & Figure S1 [file 41598_2017_4592_MOESM1_ESM.pdf]

Karst Tiankengs as refugia for indigenous tree flora amidst a degraded landscape in southwestern China

Yuqiao Su, Qiming Tang, Fuyan Mo & Yuegui Xue*
College of Life Sciences, Guangxi Normal University, Guilin 541006, Guangxi, China

***Corresponding author**
Yuegui Xue (Y.X.): xueyuegui@mailbox.gxnu.edu.cn

Supplementary information

Supplementary Table S1: Checklist of tree species ≥ 10 cm diameter at breast height and their abundance by habitat from the study. Habitat code: CDTK = Chuandong Tiankeng; SMTK = Shenmu Tiankeng; FORE = Forest Remnants; FENG = Fengshui Woods.

Supplementary Figure S1: Species-level two-way cluster dendrogram. For species codes, see Supplementary Table S1.

Supplementary Table S1. Checklist of tree species ≥ 10 cm diameter at breast height and their abundance by habitat from the study. Habitat code: CDTK = Chuandong Tiankeng; SMTK = Shenmu Tiankeng; FORE = Forest Remnants; FENG = Fengshui Woods.

Family	SpID	SpCode	Species	Abundance by habitat			
				CDTK	SMTK	FORE	FENG
Aceraceae	1	ACECHI	*Acer chingii*	0	4	0	0
Aceraceae	2	ACECOR	*Acer cordatum* var. *microcodatum*	3	0	0	0
Aceraceae	3	ACETON	*Acer tonkinense subsp. kwangsiense*	0	18	0	2
Lauraceae	4	ACTKWE	*Actinodaphne kweichowensis*	5	3	1	0
Simarubaceae	5	AILALT	*Ailanthus altissima*	2	0	0	0
Ulmaceae	6	APHASP	*Aphananthe aspera*	0	1	0	0
Cornaceae	7	AUCHIM	*Aucuba himalaica* var. *himalaica*	0	0	3	0
Lauraceae	8	BEIINT	*Beilschmiedia intermedia*	0	0	0	1
Lauraceae	9	BEIKWE	*Beilschmiedia kweichowensis*	10	8	0	0
Betulaceae	10	BETALN	*Betula alnoides*	0	0	26	0
Betulaceae	11	BETAUS	*Betula austrosinensis*	0	3	0	0
Betulaceae	12	BETLUM	*Betula luminifera*	7	1	0	0
Moraceae	13	BROPAP	*Broussonetia papyrifera*	1	0	0	0
Corylaceae	14	CARPOL	*Carpinus polyneura*	3	3	0	0
Corylaceae	15	CARPUB	*Carpinus pubescens*	0	0	8	13
Fagaceae	16	CASCHI	*Castanopsis chinensis*	0	0	0	3
Ulmaceae	17	CELBIO	*Celtis biondii*	5	5	0	1
Ulmaceae	18	CELCER	*Celtis cerasifera.*	0	0	7	8
Ulmaceae	19	CELTET	*Celtis tetrandra* ssp. *Sinensis*	0	2	0	0
Rosaceae	20	CERCAM	*Cerasus campanulata*	1	3	0	5
Rosaceae	21	CERPSE	*Cerasus pseudocerasus*	0	0	4	14
Anacardiaceae	22	CHOAXI	*Choerospondias axillaris*	5	6	6	7
Lauraceae	23	CINBUR	*Cinnamomum burmanii*	0	4	0	0
Lauraceae	24	CINSAX	*Cinnamomum saxatile*	2	7	1	2
Rutaceae	25	CLAEMA	*Clausena emarginata*	4	1	0	0
Verbenaceae	26	CLECYR	*Clerodendrum cyrtophyllum* var. *cyrtophyllum*	0	0	6	0
Cornaceae	27	CORCON	*Cornus controversa*	0	0	12	2
Coriariaceae	28	CORNEP	*Coriaria nepalensis*	0	0	8	0
Lauraceae	29	CRYCON	*Cryptocarya concinna*	7	5	0	0
Fagaceae	30	CYCARG	*Cyclobalanopsis argyrotricha*	2	0	0	0
Fagaceae	31	CYCAUS	*Cyclobalanopsis austroglauca*	5	0	0	0
Fagaceae	32	CYCGLA	*Cyclobalanopsis glauca*	7	10	0	68
Fagaceae	33	CYCJEN	*Cyclobalanopsis jenseniana*	4	2	0	0
Araliaceae	34	DENDEN	*Dendropanax dentiger*	0	4	0	0
Ebenaceae	35	DIOMOR	*Diospyros morrisiana*	1	0	0	0
Elaeocarpaceae	36	ELAJAP	*Elaeocarpus japonicus*	2	4	0	0

Family	SpID	SpCode	Species	Abundance by habitat			
				CDTK	SMTK	FORE	FENG
Juglandaceae	37	ENGROX	*Engelhardia roxburghiana*	5	4	0	0
Celastraceae	38	EUODIE	*Euonymus dielsianus*	0	1	0	0
Celastraceae	39	EUOECH	*Euonymus echinatus*	0	2	0	0
Celastraceae	40	EUOMYR	*Euonymus myrianthus*	0	0	0	1
Staphyleaceae	41	EUSJAP	*Euscaphis japonica*	0	2	0	0
Moraceae	42	FICFIS	*Ficus fistulosa*	4	0	0	0
Moraceae	43	FICHEN	*Ficus henryi*	3	0	0	0
Sapindaceae	44	HANBOD	*Handeliodendron bodinieri*	8	15	11	13
Cornaceae	45	HELJAP	*Helwingia japonica*	0	2	0	0
Rhamnaceae	46	HOVACE	*Hovenia acerba*	0	0	0	2
Aquifoliaceae	47	ILEMAC	*Ilex macrocarpa* var. *longipedunculata*	0	5	0	0
Escalloniaceae	48	ITEYUN	*Itea yunnanensis*	0	0	0	4
Juglandaceae	49	JUGREG	*Juglans regia*	0	0	0	6
Rosaceae	50	LAUFOR	*Laurocerasus fordiana*	0	0	1	0
Oleaceae	51	LIGLUC	*Ligustrum lucidum*	0	2	0	0
Lauraceae	52	LINGLA	*Lindera glauca*	9	6	0	0
Lauraceae	53	LINMEG	*Lindera megaphylla*	0	0	6	0
Lauraceae	54	LINPUL	*Lindera pulcherrima* var. *hemsleyana*	0	1	5	5
Lauraceae	55	LITCUB	*Litsea cubeba*	0	0	7	0
Lauraceae	56	LITELO	*Litsea elongata*	8	5	0	0
Fagaceae	57	LITMEG	*Lithocarpus megalophyllus*	0	1	0	0
Fagaceae	58	LITSIL	*Lithocarpus silvicolarum*	5	4	0	0
Lauraceae	59	MACCAL	*Machilus calcicola*	12	11	0	0
Lauraceae	60	MACCHI	*Machilus chinensis*	1	2	0	0
Lauraceae	61	MACGLA	*Machilus glaucifolia*	5	15	0	0
Lauraceae	62	MACMUL	*Machilus multinervia*	0	0	63	18
Lauraceae	63	MACORE	*Machilus oreophila*	2	1	0	0
Olacaceae	64	MALOLE	*Malania oleifera*	0	2	0	0
Euphorbiaceae	65	MALPHI	*Mallotus philippinensis*	0	0	0	1
Magnoliaceae	66	MANARO	*Manglietia aromatica*	3	5	0	0
Araliaceae	67	METDAV	*Metapanax davidii*	0	3	0	3
Magnoliaceae	68	MICMAR	*Michelia martinii*	0	3	0	0
Lauraceae	69	NEOCON	*Neolitsea confertifolia*	5	5	0	0
Rhamnaceae	70	PALHEM	*Paliurus hemsleyanus*	0	5	0	0
Rosaceae	71	PHOBEA	*Photinia beauverdiana*	2	4	0	0
Lauraceae	72	PHOBOU	*Phoebe bournei*	0	0	1	0
Lauraceae	73	PHONAN	*Phoebe nanmu*	2	3	0	0
Pinaceae	74	PINMAS	*Pinus massoniana*	2	0	0	0
Pittosporaceae	75	PITPUL	*Pittosporum pulchrum*	0	0	0	1
Juglandaceae	76	PLALON	*Platycarya longipes*	16	20	28	12

Family	SpID	SpCode	Species	Abundance by habitat			
				CDTK	SMTK	FORE	FENG
Pinaceae	77	PSEBRE	*Pseudotsuga brevifolia*	21	4	0	2
Fagaceae	78	QUEPHI	*Quercus phillyraeoides*	9	2	0	0
Anacardiaceae	79	RHUCHI	*Rhus chinensis* var. *chinensis*	0	0	19	0
Euphorbiaceae	80	SAPROT	*Sapium rotundifolium*	2	0	0	0
Araliaceae	81	SCHBRE	*Schefflera brevipedicellata*	15	14	0	0
Olacaceae	82	SCHJAS	*Schoepfia jasminodora*	0	2	0	0
Araliaceae	83	SCHMIN	*Schefflera minutistellata*	0	0	1	0
Sapotaceae	84	SINPED	*Sinosideroxylon pedunculatum*	3	2	0	0
Sterculiaceae	85	STELAN	*Sterculia lanceolata*	0	0	0	2
Symplocaceae	86	SYMSUM	*Symplocos sumuntia*	3	0	0	0
Myrtaceae	87	SYZBUX	*Syzygium buxifolium* var. *buxifolium*	4	0	0	0
Linaceae	88	TIROVO	*Tirpitzia ovoidea*	0	0	0	1
Meliaceae	89	TOOSIN	*Toona sinensis*	0	2	6	5
Anacardiaceae	90	TOXSUC	*Toxicodendron succedaneum* var. *succedaneum*	2	0	0	0
Staphyleaceae	91	TURMON	*Turpinia montana*	0	0	0	7
Ulmaceae	92	ULMPAR	*Ulmus parvifolia*	1	4	0	0
Caprifoliaceae	93	VIBCYL	*Viburnum cylindricum*	0	0	1	1
Caprifoliaceae	94	VIBPRO	*Viburnum propinquum*	6	0	0	0
Caprifoliaceae	95	VIBTER	*Viburnum ternatum*	0	1	0	0
Rubiaceae	96	WENUVA	*Wendlandia uvariifolia*	0	4	0	0
Total				234	258	231	210

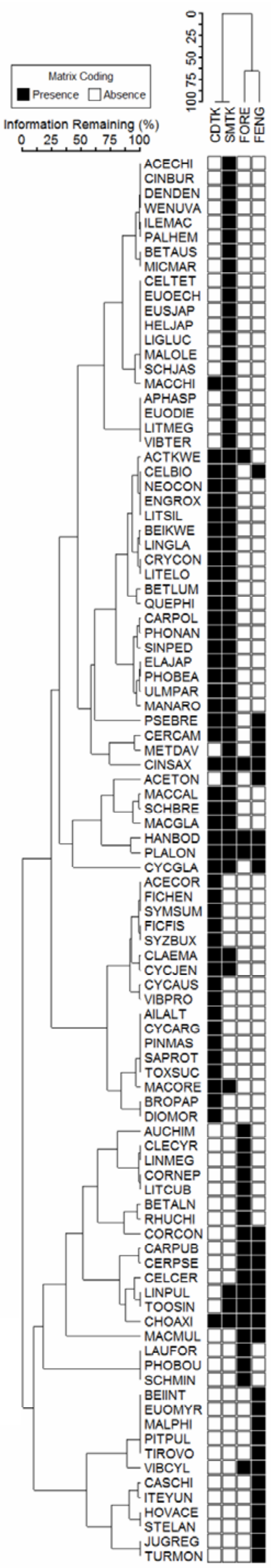

Supplementary Figure S1: Species-level two-way cluster dendrogram. For species codes, see Supplementary Table S1.
